# Chemotherapy protocols and incidence of oral mucositis. An integrative review

**DOI:** 10.1590/S1679-45082018RW4007

**Published:** 2018-04-06

**Authors:** Marina Curra, Luiz Alberto Valente Soares, Manoela Domingues Martins, Paulo Sérgio da Silva Santos

**Affiliations:** 1 Universidade de Caxias do Sul, Caxias do Sul, RS, Brazil; 2 Hospital das Clínicas, Faculdade de Medicina, Universidade de São Paulo, São Paulo, SP, Brazil; 3 Faculdade de Odontologia, Universidade Federal do Rio Grande do Sul, Porto Alegre, RS, Brazil; 4 Faculdade de Odontologia de Bauru, Universidade de São Paulo, Bauru, SP, Brazil

**Keywords:** Stomatitis/drug therapy, Stomatitis/chemically induced, Toxicity, Estomatite/tratamento farmacológico, Estomatite/induzido quimicamente, Toxicidade

## Abstract

This review aimed to analyze the scientific production on severity of oral mucositis as an adverse effect of chemotherapy. To this end, we performed a search at PubMed databases combining the keywords “oral mucositis” and “chemotherapy protocol”. To describe the investigation, the following variables were considered: journal, year/place, study design, sample, protocol used and incidence of oral mucositis. A total of 547 articles were retrieved, of which 26 were selected. Out of these 26, only 2 reported severity of oral mucositis; the others only reported the presence of the condition. Protocols for treating different types of carcinoma were evaluated in 16 (61.53%) studies, for hematological malignancies in 6 (23.07%), and for hematopoietic stem cell transplantation in 4 (15.4%). Protocols for hematopoietic stem cell transplantation entail a high risk for oral mucositis, just as chemotherapy with cytarabine and high-dose 5-fluorouracil, alkylating agents and platinumbased compounds. To provide the best prevention and treatment for oral mucositis, it is essential to know the chemotherapy protocols used and their effects on the oral cavity.

## RESUMO

Esta revisão teve como objetivo analisar a produção científica sobre a gravidade da mucosite oral como efeito adverso da quimioterapia. Para tal, nos bancos de dados do PubMed, foi realizada uma busca com a associação dos descritores “*oral mucositis*” com “*chemotherapy protocol*”. Para descrição da investigação, foram consideradas como variáveis: periódico, ano/local, delineamento da pesquisa, amostra, protocolo utilizado e incidência de mucosite oral. Foram analisados 547 artigos e, destes, 26 foram selecionados. Destes 26, apenas 2 tinham como objetivo avaliar a gravidade de mucosite oral; nos outros, a mucosite oral foi apenas relatada. Protocolos para tratamento de diferentes tipos de carcinoma foram avaliados em 16 (61,53%) estudos, para neoplasias hematológicas, em 6 (23,07%), e para transplante de células tronco hematopoiéticas em 4 (15,4%). Protocolos para transplante de células tronco hematopoiéticas são de alto risco para o desenvolvimento de mucosite oral, da mesma forma que os quimioterápicos citarabina e 5-fluorouracil em altas doses, agentes alquilantes e compostos derivados da platina. A fim de oferecer prevenção e tratamento mais adequados para mucosite oral, é imprescindível que se conheçam os protocolos quimioterápicos utilizados e seus efeitos sobre a cavidade oral.

## INTRODUCTION

The primary objective of oncological treatments, such as chemotherapy, is to destroy cancer cells. However, most chemotherapeutic agents do not act selectively, *i.e* particularly fast-growing cells, such as gastrointestinal, capillary, and immune cells.^(^ ^1^ ^-^ ^3^ ^)^ The toxicity of chemotherapeutic agents is known to be associated with the mode of action, dose and interaction between different agents in a given protocol.^(^ ^4^ ^,^ ^5^ ^)^

Oral mucositis (OM) is an important adverse effect seen in cancer patients on chemotherapy and/or radiation therapy for the head and neck.^(^ ^2^ ^,^ ^6^ ^,^ ^7^ ^)^ This condition presents clinically as erosive and/or ulcerative lesions that can cause mild to severe pain.^(^ ^1^ ^,^ ^2^ ^,^ ^8^ ^)^ These lesions usually lead to a significant decrease in quality of life, since they can prolong hospital stay, affect the nutritional status of the patient, increase the risk of infections, and increase the prescription of opioids.^(^ ^9^ ^-^ ^11^ ^)^

For these reasons, treatment of OM is extremely necessary, with the aim of relieving symptoms, accelerating tissue repair and controlling infections of oral origin. Currently, efforts are focused on preventing OM. The Mucositis Study Group of the Multinational Association of Supportive Care in Cancer and the International Society of Oral Oncology (MASCC/ISOO) has published guidelines^(^ ^12^ ^)^ to orient the clinical practice for prevention/treatment of this condition. In these guidelines, preventive measures for OM are described without specification of the chemotherapy protocol used.

The known association between toxicity and the chemotherapy protocol used may guide selection of the right prevention methods to control OM in highrisk populations.

## OBJECTIVE

To review the scientific production on severity of oral mucositis as an adverse effect of different chemotherapy protocols.

## METHODS

An integrative review with data gathered from multiple articles to assist in understanding how different chemotherapy protocols can trigger mild or severe degrees of OM. In the field of health, the amount of information is ever growing, and evidencebased practice has become a must. In this way, the methodology of integrative reviews aims to synthesize knowledge to be incorporated in the practice by identifying independent studies on the same subject and analyzing their results.

This review was based on a search at PubMed databases. The search was started and completed in May 2016. Studies published in English, Spanish or Portuguese were selected for analysis. There were no restrictions regarding the year of publication and the studies retrieved were published before May 2016. After the search, the articles were selected according to their inclusion and exclusion criteria. The search strategy was based on a combination of the keywords “oral mucositis” and “chemotherapy protocols”.

The inclusion criteria were original articles with open access to the full text, whose study subjects were patients diagnosed with OM after chemotherapy. Case reports, clinical trials, and literature reviews were excluded, as well as articles that did not describe the outcomes of the chemotherapy-associated OM.

### Study identification, selection and inclusion

Data collection was carried out by an independent researcher. After applying the search strategy with the keywords defined, the articles were selected. First the titles were read, and those that clearly did not meet the inclusion criteria for this review were readily excluded. Next, the abstracts of the selected articles were read and just like we did with the titles, the articles that clearly did not meet the predefined inclusion criteria of this review were excluded. Finally, all studies not excluded after these first two steps were read in full, and finally selected those to be included in this review.

Key data for each article were collected and entered into the database of the software Microsoft^®^ Excel^®^ for Mac 2011. The variables considered were journal, year/place, design, sample, protocol used and incidence of OM.

## RESULTS

Using the keywords of choice, 547 articles were retrieved. Of those, 325 articles were excluded by the title, 173 by the abstract and 23 by the full text, which left us with 26 articles.

The 26 articles selected were in English, published between 1987 and 2015, and the largest number of studies was from the last 5 years (n=8). All articles were original, including 24 prospective and only two retrospective studies. Of the 26 articles, only two aimed to evaluate severity of OM triggered by a given chemotherapy protocol. The other 24 studies focused on treatment efficacy and safety, and just reported the incidence of OM.

Table 1 displays the sample of each study, the chemotherapy protocol used and the severity of OM developed in each protocol. The median number of subjects assessed in each article was 135 (minimum 16 and maximum 716). The mean age of participants was 43.54 years (minimum 5.2 years and maximum 65 years).

**Table 1:** Distribution of protocols used in each study and severity of oral mucositis

Article	Sample	Protocol	Severity of mucositis
Chibaudel et al.,(13)	42 patients Mean age: 56 years Oropharyngeal carcinoma	Cetuximab (IV) D1, 8 and 15 Cisplatin D1, 8 and 15 5-FU 750mg/m^2^ D1-5	G0 = 27 (65.8%) G1 = 5 (12.2%) G2 = 4 (9.8%) G3 = 5 (12.2%) G4 = 0
Bano et al.,(14)	38 patients Mean age: 61 years Colorectal carcinoma Protocol 1 = 13 patients Protocol 2 = 12 patients Protocol 3 = 5 patients Protocol 4 = 8 patients	Protocol 1 - FOLFOX 4 - Oxaliplatin 85mg/m^2^ (IV) D1 - 5-FU 400mg/m^2^ IV bolus + 600mg/m^2^ continuous D1 and 2 - Leucovorin 200mg/m^2^ D1 and 2 Protocol 2 - FOLFOX 6 - Oxaliplatin 100mg/m^2^ (IV) D1 - 5-FU 400mg/m^2^ IV bolus + 2,400mg/m^2^ continuous D1 and 2 - Leucovorin 400mg/m^2^ D1 and 2 Protocol 3 - mFOLFOX 6 - Oxaliplatin 100mg/m^2^ (IV) D1 - 5-FU 2,000mg/m^2^ continuous D1 and 2 - Leucovorin 100mg/m^2^ D1 and 2 Protocol 4 - FOLFOX 7 - Oxaliplatin 130mg/m^2^ (IV) D1 - 5-FU 2,400mg/m^2^ continuous D1 and 2 - Leucovorin 400mg/m^2^ D1 and 2	Protocol 1 - G0 = 24% G1 and 2= 65% G3 and 4 = 11% Protocol 2 - G0 = 38% G1 and 2 = 56% G3 and 4 = 6% Protocol 3 - G0 = 21% G1 and 2 = 79% G3 and 4 = 0 Protocol 4 - G0 = 7% G1 and 2 = 90% G3 and 4 = 3%
Wang et al.,(15)	65 patients Mean age: not informed Oropharyngeal and hypopharyngeal carcinoma	Cisplatin 50mg/m^2^ (IV) D1 Tegafur 800mg (PO) D1-14 Leucovorin 60mg (PO) D1-14	G0 = 3.1% G1 = 1.5% G2 = 15.4% G3 = 73.8% G4 = 6.2%
Aapro et al.,(16)	1. Renal carcinoma = 274 patients 2. Neuroendocrine tumors = 204 patients 3. Breast cancer = 482 patients Mean age: not informed	Everolimus	1. G1-G4 = 44% 2. G1-G4 = 64% 3. G1-G4 = 59%
Tao et al.,(17)	34 patients Mean age: 16 years Nasopharyngeal carcinoma	Cisplatin 5-FU Radiation therapy	G0 = 0 G1 = 10 (29.4%) G2 = 14 (41.2%) G3 = 9 (26.5%) G4 = 1 (2.9%)
Lin et al.,(18)	124 patients Mean age: 42 years Nasopharyngeal carcinoma	3 cycles 5-FU 750mg/m^2^ D1-5 Radiation therapy	1. G0 = 82 G1 and 2 = 42 G3 and 4 = 0 2. G0 = 58 G1 and 2 = 43 G3 and 4 = 14 3. G0 = 0 G1 and 2 = 64 G3 and 4 = 60
Iwata et al.,(19)	51 patients Mean age: 55 years Breast cancer	Lapatinib 1,250mg qd Capecitabine 1,000mg/m^2^ bid	G0 = 30 (59%) G1 = 21 (41%) G2 = 0 G3 = 0 G4 = 0
Palappallil et al.,(20)	1. 50 patients 2. 50 patients Mean age: 45 (20-70) years Breast cancer	1. FAC 5-FU 500mg/m^2^ Doxorubicin 50mg/m^2^ Cyclophosphamide 500mg/m^2^ 2. AC-P Doxorubicin 60mg/m^2^ Cyclophosphamide 600mg/m^2^ Paclitaxel 175mg/m^2^	1. G1-G4 = 45 (90%) 2. G1-G4 = 2 (4%)
Baird et al.,(21)	1. 200 patients 2. 200 patients Mean age: 65 years Colorectal carcinoma	1. CAPOX 2000 Capecitabine 2,000mg/m^2^ D1-14 Oxaliplatin 130mg/m^2^ D1 2. CAPOX 1.700 Capecitabine 1,700mg/m^2^ D1-14 Oxaliplatin 130mg/m^2^ D1 Cetuximab	1. G1 = 22 (11%) G2 = 5 (2.5%) G3 = 0 (0%) G4 = 0 (0%) 2. G1 = 22 (11%) G2 = 5 (2.5%) G3 = 2 (1%) G4 = 0 (0%)
Lee et al.,(22)	32 patients Mean age: 53 years Colorectal carcinoma	Capecitabine 2,000mg/m^2^ D1-14 Cisplatin 60mg/m^2^ D1	G1-G2 = 4 (12.9%) G3-G4 = 1 (3.2%)
Abromowitch et al.,(23)	1. 85 patients 2. 82 patients 3.1. 76 patients 3.2. 73 patients 3.3. 70 patients 3.4. 68 patients 3.5. 65 patients 3.6. 65 patients Mean age: 5.1 years Lymphoblastic leukemia	1. Induction Vincristine 1.5mg/m^2^ D0, 7 and 14 Daunomycin 60mg/m^2^ D1 and 2 Cyclophosphamide 1,200mg/m^2^ D0 L-Asparaginase 2. Consolidation Vincristine 1.5mg/m^2^ D0 and 7 Cytarabine 2,000mg/m^2^ D0 and 1 Etoposide 200mg/m^2^ D0 and 1 Tioguanine 300mg/m^2^ D0-3 Methotrexate 1,000mg/m^2^ (IV) 3. Maintenance Cyclophosphamide 1,200mg/m^2^ D0 Tioguanine 300mg/m^2^ D0-3 Vincristine 15mg/m^2^ D14 and 21 Doxorubicin 30mg/m^2^ D14 Cyclophosphamide 1,200mg/m^2^ D0 Vincristine 15mg/m^2^ D28 Methotrexate 1,000mg/m^2^ (IV)	1. Induction G1-G4 = 0 (0%) 2. Consolidation G1-G4 = 5 (6.1%) 3. Maintenance 3.1 G1-G4 = 3 (3.9%) 3.2 G1-G4 = 6 (8.2%) 3.3 G1-G4 = 2 (2.9%) 3.4 G1-G4 = 3 (4.4%) 3.5 G1-G4 = 1 (1.5%) 3.6 G1-G4 = 4 (6.2%)
Shin et al.,(24)	17 patients Mean age: 38 years Hemophagocytic lymphohistiocytosis	CHOP Cyclophosphamide 750mg/m^2^ D1 Doxorubicin 50mg/m^2^ D1 Vincristine 1.4mg/m^2^ D1 Prednisone 40mg/m^2^ D1-5	G0 = 10 (58.84%) G1 = 2 (11.76%) G2 = 2 (11.76%) G3 = 3 (17.64%) G4 = 0
Blijlevens et al.,(25)	1. 109 patients with multiple myeloma Mean age: 56.8 years 2. 88 patients with non-Hodgkin's lymphoma Mean age: 50 years	1. Melphalan 200mg/m^2^ 2. BEAM Carmustine 300mg/m^2^ Etoposide 800mg/m^2^ Cytarabine 800-1,600mg/m^2^ Melphalan 140mg/m^2^	1. G0 = 10% G1-G2 = 44 % G3-G4 = 46% 2. G0 = 17% G1-G2 = 41% G3-G4 = 42%
Fadda et al.,(26)	231 patients before HSCT Mean age: 7.6 years	Alkylating agents 1. Melphalan 2. Busulfan 3. Others	1. Melphalan G0 = 20 G1-G4 = 24 2. Busulfan G0 = 34 G1-G4 = 153 3. Others G0 = 42 G1-G4 = 64
Castagna et al.,(27)	1. 29 patients 2. 24 patients Mean age: 31 years HSCT Hodgkin's lymphoma	1. Melphalan 200mg/m^2^ D1 2. BEAM Carmustine 300mg/m^2^ D1 Etoposide 800mg/m^2^ D1-4 Cytarabine 1,600mg/m^2^ D1-4 Melphalan 140mg/m^2^ D1	1. Melphalan - G3-G4 = 17 (60%) 2. BEAM - G3-G4 = 12 (50%)
Oh et al.,(28)	43 patients Mean age: 55 years Gastric carcinoma	Docetaxel 70mg/m^2^ 5-FU 1200mg/m^2^ Cisplatin 40mg/m^2^	G1 = 1 (2.3%) G2 = 1 (2.3%) G3-G4 = 0 (0)
Schmid et al.,(29)	93 patients 1. 48 2. 45 Mean age: 1. 29 years 2. 25 years Breast cancer	1. AT Doxorubicin 60mg/m^2^ Paclitaxel 200mg/m^2^ 2. HDCT Mitoxantrone 45mg/m^2^ Cyclophosphamide 2,400mg/m^2^ Etoposide 2,500mg/m^2^	1. AT - G3-G4=4.6% 2. HDCT - G3-G4 = 26.3%
Saini et al.,(30)	716 patients Mean age: 63 years Colorectal carcinoma	1. 5-FU 300mg/m^2^ IV continuous 2. 5-FU 425mg/m^2^ bolus D1-5	1. G3-G4 = 3.6% 2. G3-G4 = 19.6%
Kremens et al.,(31)	20 patients Mean age: 5.67 years Wilms tumor	Melphalan 200mg/m^2^ D1-5 Etoposide Carboplatin	G3-G4 = 10 (50%)
Lacayo et al.,(32)	38 patients 1. 15 2. 23 Mean age: not informed Acute myeloid leukemia	1. DAT Daunomycin 45mg/m^2^ D1-3 Cytarabine 100mg/m^2^ D1-7 Tioguanine 100mg/m^2^ D1-7 2. DAT + high doses cytarabine DAT Cytarabine 1,000mg/m^2^ bid D1-7	1. DAT - G3-G4 = 2 (13%) 2. DAT + high doses cytarabine G3-G4 = 8 (35%)
Leblond et al.,(33)	1. 46 patients 2. 46 patients Mean age: 64 years Waldenström macroglobulinemia	1. FAMP Fludarabine 25mg/m^2^ D1-5 2. CAP Cyclophosphamide 750mg/m^2^ D1 Doxorubicin 25mg/m^2^ D1 Prednisone 40mg/m^2^ D1-5	1. FAMP - G1-G4=0 2. CAP - G1-G4=10
Lissoni et al.,(34)	49 patients Mean age: 53.7 years Endometrial adenocarcinoma	Epirubicin 70mg/m^2^ Paclitaxel 175mg/m^2^ Cisplatin 70mg/m^2^	G1 = 12 G2 = 0 G3 = 0 G4 = 0
Wang et al.,(35)	16 patients Mean age: 42.7 years Refractory non-Hodgkin's lymphoma	Cytarabine 3,000mg/m^2^ bid D1-4 Mitoxantrone 6mg/m^2^ D1-5	G1 = 6 G2 = 2 G3 = 5 G4 = 1
Anderson et al.,(36)	148 patients 1. Bolus = 78 2. Infusion = 70 Mean age: 61 years Small cell lung cancer	Doxorubicin 35mg/m^2^ D1 Etoposide 100mg/m^2^ D1-5 Ifosfamide 5000mg/m^2^ D1	1. Bolus - G1-G4 = 24 (31%) 2. Infusion - G1-G4 = 37 (53%)
Bishop et al.,(37)	274 patients Mean age: not informed Acute non-lymphocytic leukemia	Daunorubicin 50mg/m^2^ D1-3 Etoposide 75mg/m^2^ D1-7	G3-G4 = 26%
Clavel et al.,(38)	1. 93 patients 2. 92 patients Mean age: 57.5 years Head and neck cancer	1. CABO Cisplatin 50mg/m^2^ D4 Methotrexate 40mg/m^2^ D1 and 15 Vincristine 2mg/m^2^ D1, 8 and 15 Bleomycin 10mg/m^2^ D1, 8 and 15 2. ABO Methotrexate 40mg/m^2^ D1, 8 and 15 Vincristine 2mg/m^2^ D1, 8 and 15 Bleomycin 10mg/m^2^ D1, 8 and 15	1. G3-G4 = 24% 2. G3-G4 = 31

IV: intravenous; D: day; 5-FU: 5-fluorouracil; G: grade; FOLFOX: oxaliplatin, 5-fluorouracil and leucovorin; mFOLFOX: FOLFOX with different dosage; PO: *per oris*; FAC: 5-FU, doxorubicin and cyclophosphamide; ACP: doxorubicin, cyclophosphamide and paclitaxel; CAPOX: capecitabine and oxiplatin; CHOP: cyclophosphamide, doxorubicin, vincristine and prednisone; BEAM: carmustine, etoposide, cytarabine and melphalan; HSCT: hematopoietic stem cell transplantation; AT: doxorubicin and paclitaxel; HDCT: mitoxantrone, cyclophosphamide and etoposide; DAT: daunorubicin, cytarabine and tioguanine; FAMP: fludarabine; CABO: cisplatin, methotrexate, vincristine and bleomycin; ABO: methotrexate, vincristine and bleomycin.

**Figure 1:**
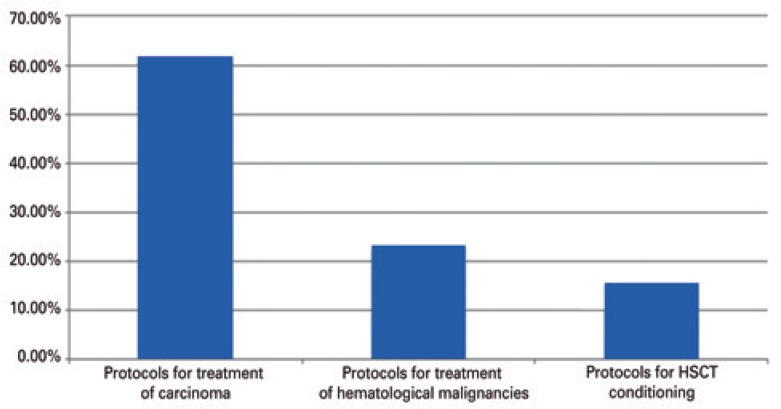
Distribution of articles according to type of treatment HSCT: hematopoietic stem cell transplantation.

Most of the studies looked at protocols for treating carcinoma 16 (61.53%), 6 (23.07%) addressed therapy for hematological malignancies and 4 (15.4%) for hematopoietic stem cell transplantation (HSCT) (Figure 1).

In the carcinoma protocols, treatments with chemotherapeutic agents, such as 5-fluorouracil (5-FU) and platinum derivatives lead to more frequent and severe cases of OM between cycles – however, numbers varied. The use of 5-FU was strongly associated with the development of OM, according to Palappallil et al.^(^ ^20^ ^)^ The authors demonstrated that 90% of patients developed OM after using 5-FU. Bolus administration of said agent also showed higher toxicity (19.6% of patients with OM) than continuous infusion (3.6% of patients with OM). For chemotherapeutics, such as ifosfamide and doxorubicin, greater severity of OM (53% of patients) was observed in individuals receiving continuous infusion.

Four articles (15.4%) addressed conditioning regimens for HSCT. Two of them compared the use of melphalan with the BEAM protocol (carmustine, etoposide, cytarabine and melphalan). The incidence of severe OM with melphalan was 46% in one study^(^ ^25^ ^)^ and 60% in another.^(^ ^27^ ^)^ The BEAM protocol also showed high incidence of severe OM in both studies (42% and 50%). This same incidence of MO with the use of melphalan was also verified by Kremens et al.,^(^ ^31^ ^)^ (50%) and by Fadda et al.,^(^ ^26^ ^)^ (54.4%). These two authors also found a high incidence of OM among patients receiving busulfan (81.82%).

Of the 26 articles, 6 (23.07%) reported cases of MO after treatment of hematological malignancies. During the induction phase for leukemia, no patients had OM; in the consolidation phase, 6.1% had OM; and in the maintenance phase, the highest rate was 8.2%. When assessing the chemotherapeutic agents used, we observed that 26% of patients on daunorubicin and etoposide, and 37.5% of patients on high-dose cytarabine had severe OM. Another protocol studied was cyclophosphamide, doxorubicin, vincristine and prednisone CHOP, and of the 17 patients treated, 7 (41.16%) had some degree of OM.

## DISCUSSION

Oral mucositis is an adverse effect of anticancer therapy that, in addition to causing discomfort/pain, may affect the nutritional health of patients. Thus, it is increasingly important to know how these lesions behave in order to provide effective prevention and treatment. Understanding the effects of chemotherapy protocols that lead to greater oral toxicity is therefore necessary.

Of the articles reviewed, it is interesting to note that only two focused on evaluating oral toxicity resulting from the anticancer agent of choice. Most of them aimed to show the response to oncological treatment using certain chemotherapy protocols, and reporting of OM was limited to the occurrence of the condition.

The mean age of subjects was 43.54 years. This mean age reflects the fact that most studies addressed treatments for carcinoma, which is more common in adults.

In this review, many articles described the effects of chemotherapy protocols for treatment of different types of carcinoma, particularly protocols based on the use of 5-FU and platinum derivatives (cisplatin and oxyplatin).^(^ ^13^ ^-^ ^18^ ^,^ ^20^ ^-^ ^22^ ^,^ ^28^ ^,^ ^30^ ^,^ ^34^ ^)^ In these articles, the incidence of OM varied a great deal, but we identified that patients who received cisplatin had more severe OM.^(^ ^15^ ^-^ ^17^ ^)^

In the study by Lin et al.,^(^ ^18^ ^)^ oral toxicity worsened with each subsequent cycle. Patients received three cycles of the same chemotherapy protocol, and in the first cycle, only 33.87% of patients developed OM - all cases were mild (grades 1 and 2). In the second cycle, 49.57% developed some degree of OM, and 12.18% were severe. In the third cycle, 100% of patients developed OM, of which 48.38% were grades 3 and 4. We found no evidence in the literature that OM has a cumulative effect with cycles, but in this review the increase in OM severity over the course of treatment was significant.

Palappallil et al.,^(^ ^20^ ^)^ proposed a comparison between two different protocols for treating breast carcinoma. In both protocols, patients received cyclophosphamide and doxorubicin but, in the first protocol, 5-FU was also administered. As much as 90% of patients who received 5-FU developed some degree of OM, against only 4% of the remaining patients, which corroborates the oral toxicity of 5-FU. Administration of 5-FU was studied by Saini et al.,^(^ ^30^ ^)^ in 716 patients with colorectal carcinoma. Only 3.6% of those who received continuous infusions of the antineoplastic drug had severe OM, whereas 19.6% of patients who received bolus infusions of 5-FU had severe OM, which implies that oral toxicity is higher when the agent is administered as a bolus. Anderson et al.,^(^ ^36^ ^)^ also observed the mode of administration of antineoplastic agents ifosfamide and doxorubicin. Differently from Saini et al.,^(^ ^30^ ^)^ they observed a higher incidence of OM in patients who received continuous administration (53%). Only 31% of patients who received doxorubicin and ifosfamide by bolus administration had some degree of OM.

The oral toxicity associated with conditioning regimens for HSCT is already well documented in the literature.^(^ ^3^ ^,^ ^25^ ^-^ ^27^ ^,^ ^31^ ^,^ ^39^ ^)^ Despite the high incidence and the fact that oral damage caused by treatment regimens used in HSCT has prevention and treatment protocols guided by the MASCC/ISOO,^(^ ^12^ ^)^ there are still very few studies focusing on the effects of chemotherapeutics on the oral cavity. In this review, four articles^(^ ^25^ ^-^ ^27^ ^,^ ^31^ ^)^ addressing said protocols were selected. Blijlevens et al.,^(^ ^25^ ^)^ and Castagna et al.,^(^ ^27^ ^)^ compared the use of melphalan and the BEAM protocol as conditioning regimens for HSCT. Both found a high incidence of OM in association with both protocols, however it was slightly higher in patients submitted to conditioning with melphalan. The two papers found that, for both protocols, approximately 50% of patients developed severe OM. This demonstrates the marked oral toxicity resulting from HSCT conditioning. Kremens et al.,^(^ ^31^ ^)^ also demonstrated a high incidence of severe MO (50%) in patients on HSCT conditioning with melphalan for Wilms tumor, despite the small sample of only 20 subjects. Still on HSCT conditioning, Fadda et al.,^(^ ^26^ ^)^ looked at the effects of chemotherapeutics melphalan and busulfan and found that, of the patients receiving melphalan, 54.54% had some degree of OM, as described in previous studies. And as much as 81.82% of patients who received busulfan developed OM. Alkylating agents are known to form inter-filamentous bonds with DNA, *i.e* since DNA alkylation requires more complex repair mechanisms and may even inhibit DNA replication. This group of patients is often associated with the development of OM.^(^ ^3^ ^,^ ^39^ ^)^

Few studies have demonstrated the relation between chemotherapy protocols used in hematological malignancies and OM severity. Abromowitch et al.,^(^ ^23^ ^)^ demonstrated toxicity resulting from each phase of leukemia treatment. No significant oral toxicities were reported, with no patient developing OM on induction, 6.1% presenting on consolidation, and less than 10% on maintenance phases. The incidence found by those authors is too low when compared with other studies. Bishop et al.,^(^ ^37^ ^)^ studied leukemia treatment with daunorubicin and etoposide (drugs used for induction and consolidation) and found that 26% of patients developed severe OM, *i.e* than that reported by Abromowitch et al.,^(^ ^23^ ^)^ Among studies addressing leukemia treatments, Lacayo et al.,^(^ ^32^ ^)^ assessed protocols with and without high-dose cytarabine for patients with acute myeloid leukemia. Patients who received high doses of cytarabine had significantly higher rates of severe OM. Wang et al.,^(^ ^35^ ^)^ also analyzed a chemotherapy protocol based on highdose cytarabine for treating refractory non-Hodgkin lymphoma. Although the number of patients enrolled was not very expressive (n=16), 37.5% of subjects had severe OM. This review also included another study by Shin et al.,^(^ ^24^ ^)^ with a small number of patients, assessing cyclophosphamide, doxorubicin, vincristine and prednisone (CHOP) protocol in 17 patients with hemophagocytic lymphohistiocytosis; of those, seven patients developed some degree of OM.

## CONCLUSION

Although oral mucositis affects not only the patient's quality of life but also the course of antineoplastic treatment, few studies focused on the relation between the chemotherapy protocol and the incidence of the condition. In this review, we observed there are scarce studies assessing the relation between oral mucositis and the chemotherapy protocol used.

Through this integrative review, we further corroborate what has been frequently demonstrated in the literature: protocols for hematopoietic stem cell transplantation entail a high risk for development of oral mucositis. We also found that cytarabine, high-dose 5-FU, alkylating agents and platinum-based compounds are commonly associated with the development of oral mucositis. Therefore, when using the aforementioned protocols, attention must be paid to prevention and treatment of this condition. In addition, it is clear that further investigations are needed concerning the oral toxicity of different drugs, to allow for more effective prevention of this condition.
